# Sotatercept in Pulmonary Arterial Hypertension: Molecular Mechanisms, Clinical Evidence, and Emerging Role in Reverse Remodelling

**DOI:** 10.3390/ijms27020767

**Published:** 2026-01-12

**Authors:** Ioan Tilea, Dragos-Gabriel Iancu, Ovidiu Fira-Mladinescu, Nicoleta Bertici, Andreea Varga

**Affiliations:** 1 Faculty of Medicine, George Emil Palade University of Medicine, Pharmacy, Science, and Technology of Targu Mures, 38 Dr. Gh. Marinescu St., 540142 Targu Mures, Romania; ioan.tilea@umfst.ro; 2 Pulmonary Hypertension Centre, Department of Internal Medicine II—Cardiology, County Emergency Hospital of Targu Mures, 50 Dr. Gh. Marinescu St., 540136 Targu Mures, Romania; andreea.varga@umfst.ro; 3 Doctoral School, George Emil Palade University of Medicine, Pharmacy, Science, and Technology of Targu Mures, 540142 Targu Mures, Romania; 4 Center for Research and Innovation in Personalised Medicine of Respiratory Diseases, Pulmonology University Clinic, Victor Babes University of Medicine and Pharmacy Timisoara, 2 Eftimie Murgu Square, 300041 Timisoara, Romania; mladinescu@umft.ro (O.F.-M.); bertici.nicoleta@umft.ro (N.B.); 5 Pulmonary Hypertension Treatment Unit, Centre of Expertise for Adult Rare Respiratory Diseases, Clinical Hospital of Infectious Diseases and Pneumophysiology “Dr. Victor Babes” Timisoara, 13 Gheorghe Adam St., 300310 Timisoara, Romania; 6 Faculty of Medicine in English, George Emil Palade University of Medicine, Pharmacy, Science, and Technology of Targu Mures, 38 Dr. Gh. Marinescu St., 540142 Targu Mures, Romania

**Keywords:** pulmonary arterial hypertension, sotatercept, activin signalling pathway, bone morphogenetic protein signalling, bone morphogenetic protein receptor type 2, vascular remodelling, reverse remodelling, disease-modifying therapy, endothelial dysfunction, precision medicine

## Abstract

Pulmonary arterial hypertension (PAH) is a severe, progressive vasculopathy characterized by endothelial dysfunction, medial hypertrophy, and maladaptive vascular and cardiac remodelling that ultimately leads to right-heart failure and premature death. Despite advances in vasodilator therapies targeting endothelin, nitric oxide, and prostacyclin pathways, a substantial proportion of patients fail to achieve or maintain a low-risk profile, highlighting the need for disease-modifying strategies. Dysregulation of transforming growth factor-β (TGF-β) superfamily signalling, with excessive activin and growth differentiation factor activity and impaired bone morphogenetic protein signalling, plays a central role in PAH pathobiology. Sotatercept, a first-in-class activin signalling inhibitor, restores this imbalance by selectively trapping pro-proliferative ligands, thereby addressing a key molecular driver of pulmonary vascular remodelling. Evidence from pivotal phase II and III trials—PULSAR, STELLAR, ZENITH, and HYPERION—demonstrates that sotatercept significantly improves exercise capacity, haemodynamics, and risk status when added to background therapy. This review summarises the molecular mechanisms underlying sotatercept’s therapeutic effects, synthesises the current clinical evidence, and discusses its emerging role as a disease-modifying agent capable of promoting reverse pulmonary vascular remodelling within contemporary PAH management.

## 1. Introduction

Pulmonary arterial hypertension (PAH) is a chronic, progressive, and life-threatening vasculopathy characterised by sustained elevations in pulmonary vascular resistance (PVR) arising from endothelial dysfunction, medial hypertrophy, intimal fibrosis, and distal microvascular remodelling. These changes impose an increasing haemodynamic load on the right ventricle (RV), ultimately leading to RV failure—the principal determinant of morbidity and mortality in PAH [1,2]. Haemodynamically, PAH is defined by mean pulmonary arterial pressure (mPAP) ≥ 20 mmHg, pulmonary arterial wedge pressure (PAWP) ≤ 15 mmHg, and PVR > 2 Wood units measured by right-heart catheterization and corresponds to Group 1 within the clinical pulmonary hypertension (PH) taxonomy [3,4].

Until recently, treatments used in PAH targeted three key vasodilatory pathways (endothelin, nitric oxide, and prostacyclin) which improve symptoms, functional capacity, and short-term haemodynamics but do not fully reverse the underlying fibroproliferative vascular pathology. Consequently, many patients fail to achieve or sustain a low-risk profile despite optimal combination therapy, underscoring the unmet need for disease-modifying approaches that directly address the biological drivers of pulmonary vascular remodelling [4,5,6].

Dysregulation of the transforming growth factor-β (TGF-β) superfamily—particularly an imbalance between anti-proliferative BMP/BMPR2–Smad1/5/8 and pro-proliferative activin/ActRIIA–Smad2/3 signalling—is central to PAH pathobiology [7,8,9,10]. Loss-of-function variants in BMPR2 and related genes diminish BMP signalling and favour activin-driven proliferation, impaired apoptosis, and maladaptive vascular remodelling [11,12,13].

Sotatercept (Winrevair™, Merck Sharp & Dohme LLC, Rahway, NJ, USA), an activin-signalling inhibitor, is the first biologic therapy developed specifically for PAH. Approved in 2024 by both the U.S. Food and Drug Administration (FDA) and the European Medicines Agency (EMA), sotatercept is a fusion protein (ActRIIA-Fc) that selectively traps circulating activins and growth differentiation factors (GDFs), thereby restoring the balance of activin–BMP signalling. This mechanism has the potential to reverse pathological vascular changes and improve RV–pulmonary arterial coupling, representing a shift from vasodilatory to disease-modifying therapy [14,15].

Evidence from phase II and III clinical trials (including PULSAR, STELLAR, ZENITH, and HYPERION) demonstrates that sotatercept, when added to optimised background therapy, yields significant and clinically meaningful improvements in PVR, 6 min walk distance (6MWD), N-terminal pro-B-type natriuretic peptide (NT-proBNP), WHO functional class (WHO-FC), and time to clinical worsening or death [15,16,17,18]. These findings support the integration of sotatercept into contemporary risk-based treatment algorithms as recommended by the 7th World Symposium on Pulmonary Hypertension (WSPH).

In this review, we summarise the molecular mechanisms underpinning activin–BMP axis dysregulation in PAH, critically evaluate the clinical evidence for sotatercept across the disease spectrum and discuss its emerging role as a disease-modifying therapy within modern PAH management strategies.

## 2. Pathophysiology of Pulmonary Arterial Hypertension

Pulmonary arterial hypertension is a systems-level vasculopathy in which genetic susceptibility, epigenetic reprogramming, and environmental triggers converge to produce maladaptive remodelling of the pulmonary microcirculation, increased pulmonary vascular resistance, and progressive right ventricular afterload. Although historically considered a disease of distal small pulmonary arteries (≈50–500 µm), contemporary histopathology reveals a pan-microvascular process involving arterioles, capillaries, and venules. The structural hallmarks include intimal fibrosis, medial hypertrophy, adventitial expansion, and perivascular inflammatory infiltration, which together drive luminal obliteration, increased vascular stiffness, and loss of compliance [19,20,21].

### 2.1. Genetic Architecture and Convergent Signalling

The genetic architecture of PAH is dominated by high-impact rare variants that disrupt vascular homeostasis, mechanotransduction, and ion-channel function. The most prevalent are loss-of-function mutations in the bone morphogenetic protein receptor type 2 (BMPR2) gene, found in familial and idiopathic forms [13,22]. Additional pathogenic variants across the TGF-β superfamily axis—including ACVRL1 (ALK1), ENG, and SMAD9—and genes involved in membrane architecture (CAV1), transcriptional regulation and trafficking (SOX17, ATP13A3), ion-channel activity (KCNK3), and developmental programming (TBX4) further expand the heritable spectrum and influence disease penetrance, age of onset, and phenotype expressivity [23,24,25].

Across genotypes, a reproducible signalling imbalance emerges between the anti-proliferative BMP/BMPR2–Smad1/5/8 and pro-proliferative activin/TGF-β–ActRIIA–Smad2/3 pathways. This dysregulation constitutes a central pathogenic axis that drives pulmonary arterial smooth muscle and endothelial proliferation, extracellular matrix deposition, and vasoconstrictor hyperreactivity—ultimately producing the fibro-proliferative vascular lesions characteristic of advanced PAH [22,26,27].

Emerging data suggest that this imbalance is amplified by epigenetic modifications, microRNA dysregulation, and altered shear-stress mechanotransduction, integrating genetic and environmental stimuli into a feed-forward circuit of vascular remodelling and RV strain [28,29].

These insights provide the biological rationale for novel disease-modifying strategies targeting the activin–BMP axis, such as sotatercept, which aim to restore vascular homeostasis and reverse maladaptive remodelling.

### 2.2. Epigenetic and Transcriptional Control

Disease-defining epigenetic programs—including altered DNA methylation, dysregulated histone modifiers (e.g., HDACs, BRD4), and pathogenic microRNAs (miR-21, miR-130/301, miR-210)—reprogram pulmonary vascular cells toward a synthetic, growth-addicted phenotype [28,30,31,32,33,34,35]. Network crosstalk with inflammatory NF-κB signalling and metabolic stress sensors (notably HIF-1α and mTOR) stabilises a pro-survival transcriptional landscape, while suppressed pro-apoptotic signalling sustains lesion cellularity and vascular remodelling [32,33,36,37,38]. These interconnected axes collectively maintain a feed-forward loop of vascular inflammation, metabolic stress, and proliferative remodelling, providing multiple nodal points for therapeutic intervention in PAH.

### 2.3. Endothelial Cell (EC) Injury, Dysfunction, and Endothelial-to-Mesenchymal Transition (EndMT)

Mechanical and oxidative stress, shear abnormalities, and intrinsic genetic defects produce endothelial dysfunction with reduced nitric oxide and prostacyclin bioactivity, coupled with excess endothelin-1 and serotonin signalling [39,40]. Early EC apoptosis is followed by clonal expansion of apoptosis-resistant endothelial cells, contributing to plexiform arteriopathy [41,42].

A subset of these dysfunctional ECs undergoes endothelial-to-mesenchymal transition (EndMT), characterised by loss of endothelial markers (CD31, VE-cadherin) and gain of mesenchymal features (α-SMA, vimentin), fuelling intimal fibrosis and vascular occlusion [43,44,45]. Perturbation of BMPR2 signalling and dominance of ActRIIA/Smad2/3 activity interact with Notch, Wnt/β-catenin, YAP/TAZ, and RhoA/ROCK pathways to coordinate cytoskeletal remodelling, migration, and extracellular matrix (ECM) production [29,46,47,48]. These interlinked molecular networks perpetuate vascular stiffening and irreversible remodelling, hallmarks of advanced PAH.

### 2.4. Pulmonary Artery Smooth-Muscle Cells (PASMCs), Fibroblasts, and Extracellular Matrix (ECM)

PASMCs acquire a de-differentiated, hyperproliferative, and migratory phenotype under the influence of PDGF, endothelin-1, TGF-β/activin ligands, serotonin/SERT, and pro-inflammatory cytokines, with disordered Ca^2+^ handling and ion-channel dysfunction (including KCNK3) sustaining depolarization and growth signalling. Adventitial fibroblasts and pericytes generate a stiff, pro-fibrotic ECM (collagens, elastin remodelling, lysyl oxidase activity) that amplifies mechanotransduction and perpetuates vasoconstrictor and growth responses [3,49,50].

### 2.5. Inflammation, Immunity, and Thrombosis

Perivascular immune infiltrates (macrophages, T/B lymphocytes, mast cells) secrete IL-6, IL-1β, TNF-α and chemokines that intensify proliferative signalling and EndMT; ectopic tertiary lymphoid structures and autoantibodies are variably observed. Platelet activation, tissue-factor expression, and impaired fibrinolysis contribute to microthrombotic occlusion, compounding resistance in the small vessel network [2,51].

### 2.6. Metabolic Reprogramming and Mitochondrial Dynamics

Pulmonary vascular cells display a cancer-like Warburg phenotype, with pyruvate-dehydrogenase inhibition, glycolysis/glutaminolysis upregulation, impaired fatty-acid oxidation, and dysfunctional oxidative phosphorylation. Structural mitochondrial abnormalities—excess fission (DRP1 activation), reduced fusion (e.g., MFN2 downregulation), fragmented networks, and altered cristae—drive reactive oxygen species signalling, DNA-damage responses, and apoptosis resistance. These changes are evident in-patient tissues and correlate with RV metabolic maladaptation and contractile inefficiency, linking microvascular disease to pump failure [39,43,52,53,54].

### 2.7. Hemodynamic–Ventricular Coupling and Outcomes

Progressive increases in pulmonary vascular resistance and impedance impose a chronic pressure overload on the RV, leading to maladaptive remodelling characterized by RV–arterial uncoupling, ischemia, and interstitial fibrosis [54,55]. Throughout the disease course, RV function remains the principal determinant of prognosis in PAH, serving as the integrative endpoint of upstream pulmonary microvascular pathology and systemic clinical outcomes [56,57]. The dynamic interplay between ventricular afterload, contractile adaptation, and myocardial reserve ultimately defines the transition from compensated hypertrophy to overt right heart failure, underscoring the central pathophysiological role of RV–arterial coupling in PAH progression [54,58].

**Figure 1:**
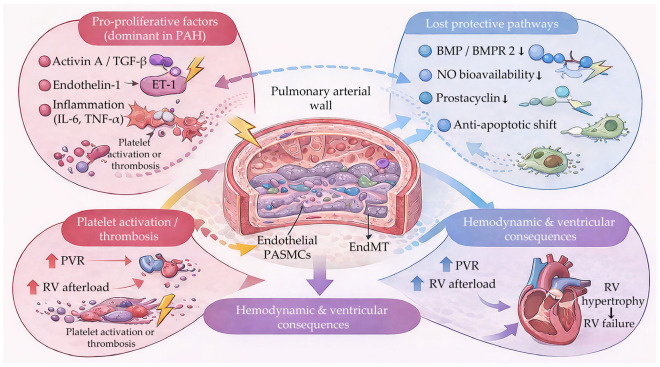
Pathophysiology of pulmonary arterial hypertension.

Pulmonary arterial hypertension is driven by a complex imbalance between pro-proliferative and protective signalling pathways within the pulmonary arterial wall. Excess activin/TGF-β and endothelin signalling, together with inflammatory and thrombotic stimuli, promote endothelial dysfunction, smooth muscle cell proliferation, extracellular matrix deposition and endothelium-to-mesenchymal transition. In parallel, loss of protective BMP/BMPR2, nitric oxide and prostacyclin signalling further amplifies vascular remodelling, leading to progressive increases in pulmonary vascular resistance, right heart failure (Figure 1).

BMP, bone morphogenetic protein; BMPR2, bone morphogenetic protein receptor type 2; EndMT, endothelial-to-mesenchymal transition; ET-1, endothelin-1; IL-6, interleukin-6; NO, nitric oxide; PAH, pulmonary arterial hypertension; PASMCs, pulmonary artery smooth muscle cells; PVR, pulmonary vascular resistance; RV, right ventricle; TGF-β, transforming growth factor beta; TNF-α, tumour necrosis factor alpha.

### 2.8. Therapeutic Implication: Targeting the Activin/BMP Axis

The multifactorial, “multihit” model of PAH pathogenesis converges on a persistently dysregulated, pro-proliferative, anti-apoptotic, and pro-fibrotic vascular phenotype sustained by an imbalance within the transforming growth factor β (TGF-β) superfamily and by interlinked inflammatory–metabolic crosstalk [7,59,60]. Sotatercept, a first-in-class activin receptor type IIA–Fc (ActRIIA-Fc) fusion protein, acts as a ligand trap for circulating activins and select growth differentiation factors (GDFs). Through this mechanism, it attenuates Smad2/3 signalling and restores the bone morphogenetic protein receptor type 2 (BMPR2)–Smad1/5/8 pathway, thereby reducing pulmonary arterial smooth muscle cell (PASMC) proliferation, mitigating endothelial cell (EC) dysfunction—including endothelial-to-mesenchymal transition (EndMT)—normalizing extracellular matrix (ECM) dynamics, and promoting reverse vascular remodelling when administered alongside established vasodilator therapies [46,61]

## 3. The Role of TGF-β in Pulmonary Arterial Hypertension

The transforming growth factor beta (TGF-β) family is significantly involved in the initiation and progression of PAH. TGF-β serves as a crucial regulator of vascular remodelling and pulmonary inflammation, as well as cardiac hypertrophy and fibrosis. Among the various receptors in the TGF-β family, bone morphogenetic receptor type 2 (BMPR2) is particularly important in the pathophysiology of PAH. The TGF-β signalling pathway and BMPR2 are essential for the development and progression of PAH. TGF-β is encoded by more than 33 genes and exists as homo- and heterodimers whose effects are context-dependent; this means that cells can demonstrate different, or even opposing, responses to TGF-β signals, depending on the situation [62]. TGF-β functions as a bidirectional regulator, capable of both stimulating and inhibiting cell proliferation. The TGF-β superfamily can be categorized into three isoforms of TGF-β, as well as activins, nodal proteins, bone morphogenetic proteins (BMPs), and growth differentiation factors (GDFs). While most TGF-β ligands are homodimers, various biologically active heterodimer combinations have also been recognized. Though there are many TGF-β ligands, the signalling pathway is primarily mediated by a smaller number of receptors and effectors known as Smad proteins [62]. The Smad family consists of eight members, categorized into three subtypes: receptor-regulated Smads (R-Smads), co-partner Smads (co-Smads), and inhibitory Smads (I-Smads) [63]. R-Smad family includes Smad1, Smad2, Smad3, Smad5, and Smad8/9 [64]. Trimers of two receptor-regulated Smads and one co-partner Smad (co-Smad) act as transcription factors that regulate the expression of certain genes [63,65]. Smad 1/5/8 mediate BMP signalling, while Smad2/3 mediate TGF-β and activin signalling. TGF-β ligands bind to type II receptors (transmembrane serine/threonine kinases receptor), which subsequently recruit and phosphorylate type I receptors. Each ligand family binds to a specific type II receptor [66], with seven types I and five type II receptors identified to date [67]. The type I receptor phosphorylates receptor-regulated Smads (R-Smads), allowing them to form complexes with co-Smads. These R-Smad/co-Smad complexes accumulate in the nucleus, functioning as transcription factors to regulate target gene expression [63]. There are three types of activins: Activin A, Activin B, and Activin AB. Activins are crucial for embryogenesis, osteogenesis, and the regulation of various hormones, including pituitary, gonadal, hypothalamic, and insulin hormones, and represent an important neuronal survival factor. Activin A specifically binds to the activin receptor type IIA (ActRIIA), which regulates critical signalling pathways for inflammation, cell proliferation, apoptosis, and tissue homeostasis.

Under normal physiological conditions, the activation balance of TGF-β and BMPR2 results in a harmonious interplay between pro-proliferative and anti-proliferative processes. However, mutations that compromise BMPR2 function trigger aberrant proliferation of endothelial and smooth muscle cells in the pulmonary arteries. Reduced activation of the BMPR2 pathway has been observed in both hereditary PAH (with >80% of these patients carrying identified mutations in the BMPR2 gene) and non-hereditary forms of PAH [68]. In contrast, increased TGF-β activity is a critical factor in the dysfunction of endothelial and smooth muscle cells in PAH-affected pulmonary arteries.

PAH is characterized by a signalling imbalance between TGF-β and BMP [69]. Activation of the BMPR2 inhibits smooth muscle cell proliferation in the pulmonary circulation, individuals with BMPR2 mutations being at an elevated risk for developing PAH [7]. Conversely, increased TGF-β signalling exerts complex and opposing effects on various tissues [70]. TGF-β superfamily’s ligands fulfil essential roles in embryonic development, angiogenesis, wound healing, inflammation, and immune T-cell functions. Disruption of TGF-β signalling can lead to inflammation, autoimmune disorders, fibrosis, cancer, or PAH [71]. In PAH, there is a reduction in antiproliferative BMP signalling, while activin A levels are increased, with activin binding to ActRIIA triggering proliferative signalling in vascular cells and promoting occlusive remodelling in the pulmonary vascular bed [71,72,73,74].

Remodelling affects all three layers of the pulmonary vascular wall. In patients with BMPR2 mutations, endothelial cell loss occurs due to destruction and enhanced susceptibility to apoptosis [2,49,51]. Intimal lesions, including eccentric intimal thickening and various forms of fibrous, plexiform, concentric, dilated, and/or angiomatoid lesions, contribute to the narrowing of small pulmonary arteries [75]. Recent studies suggest that mesenchymal transformation of endothelial cells may be driven by alterations in BMPR2, which damages the intimal layer of the pulmonary vascular wall [43,76]. Intimal fibrosis and neointima formation result from the uncontrolled proliferation of apoptosis-resistant endothelial cells, while hyperplasia of pulmonary artery smooth muscle cells leads to medial hypertrophy and fibroblast proliferation, collagen modification, and inflammatory cell infiltration lead to remodelling of the adventitia [77]. Additionally, smooth muscle cell migration stimulates the neomuscularization of previously non-muscularized arterioles, and increased platelet activation in contact with dysfunctional endothelium contributes to in situ thrombus formation. The thickening of the intimal layer is primarily due to hyperplasia and hypertrophy of smooth muscle cells [78]. Furthermore, changes in the immune system and an increase in pro-inflammatory phenomena further exacerbate vascular remodelling [4,79], leading to the progressive narrowing, occlusion, and loss of pulmonary vessels. In advanced stages of PAH, plexiform lesions develop within the adventitia of pulmonary vessels, arising from the unchecked proliferation of smooth muscle cells in the arterial wall and neoangiogenesis.

In the right heart, TGF-β signalling stimulation promotes matrix remodelling, myofibroblast proliferation and activation, and cardiac fibrosis [73,80,81]. The remodelling of pulmonary circulation adversely affects the structure and function of the right ventricle. Initially, to adapt to increased pulmonary vascular resistance, the right ventricle undergoes compensatory hypertrophy and experiences a four- to five-fold increase in contractility in response to heightened afterload [82,83]. This compensatory change includes angiogenesis, cardiomyocyte hypertrophy, and ventricular wall thickening, during which the right ventricle and pulmonary artery maintain coupling. As hypertension progresses, the right ventricle enters a maladaptive phase characterized by increased wall stress, metabolic changes, reduced capillary density, fibrosis, cellular apoptosis, inflammation, immune cell recruitment, oxidative stress, neurohormonal stress, and disruptions in the renin–angiotensin–aldosterone system. This maladaptive phase is associated with impaired ventriculo–arterial coupling, reduced ventricular contractile reserve, ischemia, elevated biomarker levels, and reduced functional capacity [84]. Ultimately, these changes lead to right ventricular systolic dysfunction and right ventricular failure [58,82,85], the main factor influencing the clinical course and survival rates of PAH patients [1,4,43,49,53,79,84,86]. Right ventricular dilation and contractility dysfunction also cause the interventricular septum to shift leftward and prolapse into the left ventricle, impairing left ventricular compliance and diastolic filling.

Taken together, these observations support a unifying pathobiological framework in which PAH is driven not only by vasoconstriction but by a profound imbalance within the TGF-β superfamily, characterised by reduced antiproliferative BMP/BMPR2 signalling and excessive activin- and TGF-β-mediated pro-proliferative activity. This signalling disequilibrium promotes pulmonary vascular remodelling, endothelial dysfunction, smooth muscle cell proliferation, inflammation, and progressive right ventricular maladaptation. Importantly, the persistence of dysregulated activin signalling despite established vasodilator therapies identifies this pathway as a rational and biologically compelling therapeutic target, providing the mechanistic basis for the development of novel disease-modifying strategies such as activin-signalling inhibition.

## 4. New Pathophysiological Pathway, New Therapy

Despite substantial progress in understanding the pathobiology of PAH and the development of multiple evidence-based therapies, this disease remains a progressive, life-limiting disease associated with unacceptably high morbidity and mortality [1,2,49,51,87]. Traditionally, treatment strategies have centred on counteracting abnormal pulmonary vasoconstriction, aiming to reduce pulmonary vascular resistance and alleviate right ventricular afterload [68,88,89,90,91,92,93,94]. These therapies modulate the three classical pathophysiological pathways:

1. Nitric oxide pathway—modulated by phosphodiesterase-5 inhibitors (sildenafil, tadalafil) and the soluble guanylate cyclase stimulator (riociguat);
2. Endothelin pathway—antagonised by endothelin receptor antagonists (ambrisentan, bosentan, macitentan);
3. Prostacyclin pathway—activated by prostacyclin analogues (epoprostenol, iloprost, treprostinil) and IP receptor agonists (selexipag).

Although these drug classes have substantially improved symptoms, haemodynamic and survival, none of them modulates dysregulated activin signalling, a key pathogenic axis in PAH.

In a landmark study published in 2023, Guignabert et al. [95] demonstrated that circulating activins serve as independent prognostic biomarkers in PAH and, importantly, that activin A levels remain largely unchanged despite treatment with mono-, dual- or triple-combination PAH therapies. This observation highlighted the persistence of dysregulated activin signalling as an unmet therapeutic target, despite optimal treatment with conventional vasodilatory agents [95].

A major paradigm shift occurred in 2024 when the U.S. Food and Drug Administration (FDA) and the European Medicines Agency (EMA) approved the first biologic therapy specifically developed for PAH: sotatercept, a recombinant homodimeric fusion protein of the human activin receptor type IIA-Fc (ActRIIA-Fc), acts as a ligand trap for members of the TGF-β superfamily, effectively removing excess activin-A and other ligands that bind to ActRIIA. By selectively binding circulating activins and growth differentiation factors (GDFs), sotatercept restores the balance between activin- and BMP-mediated signalling, suppressing proliferative pathways while reinstating antiproliferative and pro-apoptotic signals within the pulmonary arterial wall [96]. This mechanism directly targets the vascular remodelling process that underlies PAH progression and represents a major therapeutic breakthrough—introducing a new disease-modifying pathway into a field historically dominated by vasodilators.

Unlike previously available PAH therapies, which predominantly exert vasodilatory effects on the pulmonary vascular bed, sotatercept directly targets the dysregulated molecular mechanisms underlying pulmonary vascular remodelling, thereby offering the potential to modify disease progression and improve long-term prognosis.

**Figure 2:**
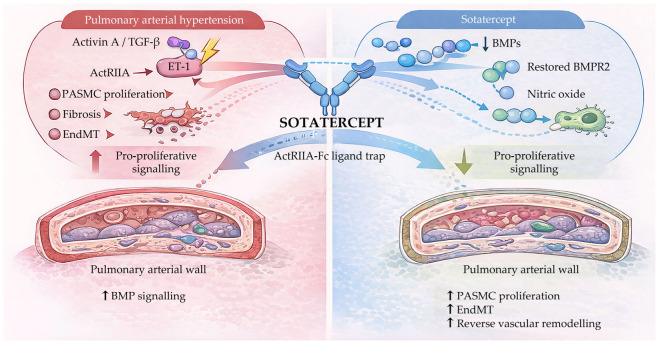
Mechanism of action of sotatercept in pulmonary arterial hypertension.

Figure 2 summarises the imbalance between pro-proliferative activin/TGF-β signalling and protective BMP/BMPR2 pathways in PAH. By acting as an activin receptor type IIA–Fc ligand trap, sotatercept restores BMP signalling, improves nitric oxide bioavailability, and reduces pathological vascular remodelling within the pulmonary arterial wall.

ActRIIA, activin receptor type IIA; ActRIIA-Fc, activin receptor type IIA–Fc fusion protein; BMP, bone morphogenetic protein; BMPR2, bone morphogenetic protein receptor type 2; EndMT, endothelial-to-mesenchymal transition; ET-1, endothelin-1; NO, nitric oxide; PASMC, pulmonary artery smooth muscle cell; PAH, pulmonary arterial hypertension; TGF-β, transforming growth factor beta.

### 4.1. Pharmacokinetics of Sotatercept

Following subcutaneous administration, sotatercept demonstrates an absolute bioavailability of approximately 66%. At the recommended dose of 0.7 mg/kg, the drug achieves a maximum plasma concentration (Cmax) of 9.7 μg/mL, with a median time to peak concentration (Tmax) of 7 days (range: 2–8 days). Repeated administration every three weeks results in a steady-state area under the concentration–time curve of 172 μg·day/mL after approximately 15 weeks, consistent with the drug’s long elimination profile.

Sotatercept has a relatively limited volume of distribution (≈5.3 L), indicating confinement largely within the intravascular compartment. As a recombinant fusion protein, it undergoes catabolic degradation into smaller peptides, like other monoclonal antibody–based therapeutics. The drug exhibits a systemic clearance of 0.18 L/day and a terminal elimination half-life of approximately 24 days, supporting the approved dosing interval of once every three weeks.

In the phase III STELLAR trial, 27% of participants developed anti-drug antibodies (ADAs); however, after 24 weeks of observation, these antibodies did not produce any clinically meaningful effects on pharmacokinetics, pharmacodynamics, efficacy, or safety outcomes [97].

The pharmacokinetic, immunogenicity, efficacy, and safety profiles of sotatercept have been extensively evaluated across multiple phase II and phase III clinical trials, forming a robust evidence base for its use in PAH.

### 4.2. Phase II Clinical Trials

The PULSAR trial was a Phase II, randomized, double-blind, placebo-controlled study designed to assess the efficacy and safety of sotatercept in patients with PAH classified as WHO-FC II or III. A total of 106 patients were randomized in a 1:1:1 ratio to receive placebo, sotatercept 0.3 mg/kg, or sotatercept 0.7 mg/kg. Study treatments (sotatercept or placebo) were administered subcutaneously every 21 days, with efficacy and safety evaluations performed at baseline and at 21-day intervals throughout the 24-week treatment period.

The primary endpoint was the change from baseline in PVR at week 24. Sotatercept treatment resulted in a significant reduction in PVR (−162.2 dyn·s·cm^−5^ in the 0.3 mg/kg group and −255.9 dyn·s·cm^−5^ in the 0.7 mg/kg group), compared with a minimal decrease (−16.4 dyn·s·cm^−5^) in the placebo group. These haemodynamic improvements were consistent across all prespecified subgroups, irrespective of background therapy intensity (mono/dual vs. triple therapy) or concomitant parenteral prostacyclin use. Data sets are presented in Table 1.

Secondary endpoints included changes from baseline to week 24 in 6MWD, NT-proBNP concentration, tricuspid annular plane systolic excursion (TAPSE), and WHO-FC, and the incidence of clinical worsening. At week 24, both sotatercept dosing groups demonstrated consistent and clinically meaningful improvements across functional, biomarker, and clinical status measures compared with placebo. Improvements in 6MWD and NT-proBNP were concordant with the reductions in PVR, while favourable shifts in WHO-FC and a lower incidence of clinical worsening further supported the therapeutic effect of sotatercept. Data are presented in Table 2.

A total of 97 patients from the PULSAR trial entered the open-label extension study (PULSAR-OLE), which further evaluated the long-term efficacy and safety of sotatercept over 18 and 24 months. Patients who transitioned from placebo to sotatercept demonstrated significant reductions in PVR at both 18 and 24 months compared with their baseline values. In parallel, improvements were identified in 6MWD, WHO-FC, and NT-proBNP concentrations in both groups (those crossed over from placebo and those who continued sotatercept therapy) [16]. Data are presented in Table 3.

Adverse events were reported in 30.8% of participants, although only 4.8% were considered treatment-related. Reported adverse events included fever, increased red blood cell count, systemic lupus erythematosus, ischemic stroke, pleural effusion, and worsening pulmonary hypertension. Telangiectasia was observed in 10.6% of patients, typically occurring about 1.5 years after the initiation of sotatercept therapy.

The SPECTRA clinical trial examined the effects of sotatercept on changes in maximal oxygen consumption in PAH patients in FC III over a 24-week period. Assessments were conducted using invasive cardiopulmonary exercise tests and cardiac magnetic resonance imaging (cMRI) to evaluate the right ventricular function [98]. At the 24-week mark, there was a significant improvement in maximal oxygen consumption, which was sustained through the 48-week follow-up. Furthermore, improvements were apparent in secondary endpoints and cMRI imaging parameters [98]. SPECTRA is the only prospective study who demonstrated a reduction in both right ventricular end-diastolic volume and RV mass, necessary criteria for considering RV reverse remodelling. The documented structural and functional improvement in the right ventricle suggest that sotatercept could be regarded as a disease-modifying agent [99].

### 4.3. Phase III Clinical Trials

The STELLAR trial was the pivotal phase III, randomized, double-blind, placebo-controlled study that supported the regulatory approval of sotatercept by both the U.S. Food and Drug Administration (FDA) and the European Medicines Agency (EMA). This study evaluated the efficacy and safety of sotatercept over a 24-week treatment period in patients with PAH in WHO-FC II–III, all receiving stable background therapy.

The primary endpoint was the change in 6MWD at 24 weeks from baseline. The trial included prespecified secondary endpoints, evaluating multicomponent clinical improvement, changes in PVR, NT-proBNP and WHO-FC, time to death or clinical worsening and risk status as per the French risk score classification, time to death or first occurrence of a clinical worsening event and patient-reported quality of life.

A total of 323 participants were randomized to receive sotatercept or placebo every three weeks on top of stable background PAH therapy. Randomization ensured balanced distribution of baseline characteristics, and the trial population reflected contemporary real-world PAH management, with most patients receiving dual or triple therapy, including parenteral prostacyclin.

At 24 weeks, the mean change from baseline in 6MWD was +40.1 m (95% CI: +29.9 to +50.2) in the sotatercept group, compared to −1.4 m (95% CI: −13.2 to +10.3) in the placebo group. For secondary endpoints, the sotatercept group exhibited statistically significant improvements across 8 of the 9 evaluated endpoints. Specifically, the risk of clinical worsening (assessed by a composite endpoint of time to first event of clinical worsening or death) was reduced by 84% in the sotatercept group compared to the placebo group.

Treatment with sotatercept resulted in significant improvements in patient-reported quality of life, reflected by gains in PAH-SYMPACT physical impact and cardiopulmonary symptom domains at week 24. However, no significant differences were found between sotatercept and placebo in the cognitive/emotional domain (*p* = 0.16) [15]. Data are presented in Table 4.

A prespecified sub-analysis of the STELLAR trial demonstrated that sotatercept reduced the risk of death or clinical worsening by 84% (HR 0.16; 95% CI, 0.08–0.35). Moreover, sotatercept reduced the risk of the most clinically meaningful worsening events (including death, hospitalization for PAH deterioration, or lung transplantation) by 88% [15] (Table 5).

In the STELLAR trial, sotatercept showed a generally favourable and manageable safety profile. Treatment adherence was high, exceeding 98% in both the sotatercept and placebo arms. Adverse events consistent with the drug’s activin-signalling mechanism—most notably epistaxis, telangiectasia, and dizziness—occurred more frequently in the sotatercept group than in the placebo group, in line with the expected on-target pharmacodynamic effects. The incidence of serious adverse events (SAEs) was lower with sotatercept (14.1%) compared with placebo (22.5%), and treatment-related SAEs were rare, occurring in only 1.2% of sotatercept-treated participants.

Predefined adverse events of special interest (AESI) occurred in 49.1% of sotatercept-treated patients compared with 36.2% of those receiving placebo. These AESIs predominantly comprised bleeding events (including epistaxis), thrombocytopenia telangiectasia, and increases in haemoglobin. These AESIs are biologically plausible in the context of activin/GDF–BMP pathway modulation, given the role of TGF-β superfamily signalling in endothelial homeostasis/angiogenesis and in haematopoiesis. Bleeding and telangiectasia may reflect enhanced superficial angiogenesis and vascular fragility resulting from alterations in endothelial TGF-β/activin signalling, whereas thrombocytopenia is consistent with reduced activin-mediated support of megakaryocyte maturation, with higher rates observed in patients receiving parenteral prostacyclin (21.5% vs. 3.1%). In parallel, haemoglobin increases are compatible with relief of inhibitory signalling on erythropoiesis. A mean platelet decline of 15.9 × 10^9^/L was observed at week 24 in the sotatercept group, compared with a slight increase in the placebo arm.

Overall, adverse events associated with sotatercept were predominantly mild to moderate in severity, and serious events were uncommon. Nevertheless, given the on-target modulation of the activin/GDF–BMP pathway, the available evidence supports the implementation of structured laboratory monitoring, particularly of haemoglobin levels and platelet counts, as well as heightened clinical vigilance in patients receiving anticoagulation or parenteral prostacyclin therapy. This predictable safety profile, when coupled with appropriate monitoring strategies, underscores the clinical manageability of sotatercept in contemporary PAH treatment paradigms [15] (Table 6).

Nine deaths were reported overall—2 (1.2%) in the sotatercept arm and 7 (4.4%) in the placebo arm—and none were considered attributable to sotatercept treatment [15].

Several post hoc analyses from the STELLAR dataset further examined the consistency of sotatercept efficacy and safety across clinically relevant subgroups. Findings presented by Meyer and colleagues demonstrated that treatment effects were preserved irrespective of background PAH therapy [100]. Similarly, analyses by Humbert et al. showed that sotatercept provided comparable benefit across different baseline risk strata risk group, while Kopec et al. confirmed that efficacy and safety were maintained regardless of the type or burden of comorbidities [101,102]. Collectively, these results highlight the robustness and broad applicability of sotatercept therapeutic profile across diverse patient populations.

The ZENITH trial was a global, phase III, randomized, double-blind, placebo-controlled study designed to evaluate the effect of sotatercept added to maximal background therapy on the time to first major morbidity or mortality event in patients with PAH at high risk of death, defined as REVEAL Lite 2.0 score ≥ 9, and in WHO-FC III or IV [103].

A total of 172 participants were enrolled and randomized in a 1:1 ratio into two groups: one group received sotatercept plus PAH-specific background therapy, while the other received a placebo alongside background therapy.

The primary endpoint was the time to first confirmed morbidity or mortality event, defined as death from any cause, lung transplantation, or hospitalization ≥ 24 h due to PAH worsening. Key secondary endpoints included: overall survival; lung-transplantation–free survival; changes from baseline at week 24 in NT-proBNP, mPAP, PVR, WHO-FC, 6MWD, REVEAL Lite 2.0 risk status, the proportion of patients improving to low or intermediate risk; and quality-of-life outcomes assessed using EQ-5D-5L instrument.

By November 2024, an independent data monitoring committee recommended early termination of the study after an interim analysis demonstrated a statistically and clinically significant reduction in morbidity and mortality with sotatercept compared to placebo [104].

Following study discontinuation, participants were offered enrolment into the long-term extension SOTERIA trial to continue or initiate sotatercept therapy [105,106]. Notably, this represented the first phase III PAH trial to be stopped early due to clear evidence of superior efficacy of an investigational treatment

The findings from ZENITH clinical study confirmed the substantial clinical benefit of sotatercept in a high-risk population; they were published on March 31 and submitted to regulatory authorities to support an extension of the drug’s indication. The study showed that at a median follow-up of 10.6 months (range, 0.3–26.1), sotatercept reduced the relative risk of major morbidity and mortality events (the composite of all cause death, lung transplantation and PAH worsening related hospitalization of ≥24 h) by 76% (HR = 024, [95%CI, 0.13–0.43]; *p* < 0.0001) versus placebo [106].

The primary composite endpoint assessed in the ZENITH Phase III trial is summarised in Table 7, whereas Table 8 provides the corresponding secondary clinical, haemodynamic, and patient-reported outcomes.

The safety profile of sotatercept in the ZENITH trial was consistent with findings from previous phase II and phase III studies, with no new or unexpected safety signals identified. Adverse events aligned with the known, mechanism-related effects of activin pathway modulation—such as manageable increases in haemoglobin, decreases in platelet counts, and mucocutaneous events—and the overall incidence and severity of adverse reactions were comparable to earlier clinical experience [17] (Table 9).

The HYPERION trial was a global, phase III, randomized, double-blind, placebo-controlled study designed to assess the efficacy of sotatercept added to background therapy in patients with newly diagnosed PAH who were at intermediate or high risk within the first year following diagnosis. Of the 441 patients screened, 320 were enrolled and randomized 1:1 to receive either sotatercept plus background therapy or placebo plus background therapy.

The primary endpoint was a composite measure of time to clinical worsening, defined as the first occurrence of death from any cause, lung transplantation, atrial septostomy, hospitalization due to PAH worsening (≥24 h), or deterioration in exercise capacity attributable to PAH. Secondary endpoints included: multicomponent improvement, risk score improvement (REVEAL Lite 2 score and Simplified French risk score), NT pro-BNP levels, improvement of the WHO-FC or maintain of FC II, improvement in 6MWD, overall survival [18,107].

Earlier in the year, following the positive interim results of the ZENITH Phase III trial and after consultation with the FDA, both the sponsor and an independent clinical data monitoring committee determined that continuing HYPERION with a placebo arm would be ethically inappropriate [104]. As a result, the study was terminated early, and participants were offered enrolment into the SOTERIA open-label extension to receive or continue receiving sotatercept [105].

Results from HYPERION were presented at the ERS Congress 2025 and published concurrently in The New England Journal of Medicine. The addition of sotatercept to background therapy within 12 months of diagnosis resulted in a 76% reduction in the risk of clinical worsening compared with placebo. Across all prespecified patient subgroups, treatment effects were consistent with the overall benefit. Notably, the Kaplan–Meier curves demonstrated an early and sustained separation beginning after the third dose, suggesting a rapid onset of clinical effect. The number needed to treat (NNT) was 5, indicating that one clinical worsening event was prevented for every five patients treated over a 12-month period [18].

Regarding safety, the profile of sotatercept in HYPERION was consistent with previous trials, with the most frequently observed adverse events being increases in haemoglobin, bleeding events (primarily epistaxis and gingival bleeding), and telangiectasia. Importantly, the rates of serious and severe adverse events were similar between the sotatercept and placebo groups, despite the longer treatment exposure in the sotatercept arm (14.6 months) compared with placebo (11.5 months). These findings reinforce the predictability and stability of the safety profile across the sotatercept clinical development program.

### 4.4. Ongoing Clinical Trials

The SOTERIA clinical trial (MK-7962-004/A011-12; NCT04796337) is an ongoing, open-label, long-term follow-up extension designed to evaluate the prolonged safety, tolerability, and efficacy of sotatercept in adults with PAH who previously completed parent sotatercept trials, including the phase 2 PULSAR and phase 3 STELLAR studies, as well as other phase 3 trials such as ZENITH and HYPERION [108,109]. Beyond safety, key secondary objectives include the assessment of long-term changes in 6MWD, NT-proBNP, PVR, WHO-FC, overall survival, and evolution of risk status according to the simplified French risk score [108,109,110]. Each participant may receive sotatercept for approximately four years, providing an opportunity to characterise durability of response and late safety signals over an extended exposure period [108,109,110].

Interim analyses from SOTERIA have shown that patients initially randomised to placebo in their parent trials and subsequently crossed over to sotatercept experienced clinically meaningful improvements over one year, with increases in 6MWD and reductions in NT-proBNP, together with a marked shift towards lower risk categories [105]. Conversely, among patients who had already been receiving sotatercept in the parent trials and continued treatment in SOTERIA, 6MWD, NT-proBNP, FC distribution and low-risk status remained stable at 24 weeks and one year, supporting the maintenance of therapeutic benefit over time [105].

The MOONBEAM trial (MK-7962-008; NCT05587712) is a phase II, open-label, single-arm study specifically designed to evaluate sotatercept in paediatric PAH. It enrols children aged 1 to <18 years with WHO Group 1 PAH on standard background therapy and focuses primarily on safety, tolerability, and pharmacokinetics over 24 weeks of treatment, with pharmacodynamic and exploratory efficacy endpoints (such as changes in exercise capacity, biomarkers and haemodynamic) collected to inform future paediatric development [111]. 

The CADENCE trial (MK-7962-007/A011-16; NCT04945460) is a phase II, double-blind, randomised, placebo-controlled study investigating sotatercept in adults with combined post- and precapillary pulmonary hypertension (Cpc-PH) secondary to heart failure with preserved ejection fraction (HFpEF). The primary endpoint is the change in PVR at 24 weeks, with 6MWD as the principal key secondary endpoint; additional outcomes include right-heart structure and function, NT-proBNP, clinical worsening events and patient-reported outcomes. Participants are randomised to placebo or sotatercept (0.3 mg/kg or an up-titrated 0.7 mg/kg regimen) administered subcutaneously every three weeks, with follow-up extending beyond the blinded treatment phase to allow evaluation of medium-term safety and efficacy in this high-risk HFpEF–Cpc-PH phenotype [112]. In November 2025, results demonstrated that the trial met its primary endpoint, with sotatercept producing a statistically significant reduction in pulmonary vascular resistance (PVR) at week 24 compared with placebo [113].

To complement CADENCE, an extension study in Cpc-PH due to HFpEF (MK-7962-023; NCT06814145, HARMONIZE) has been initiated. This phase II, double-blind extension allows eligible participants who completed CADENCE without early discontinuation to continue sotatercept treatment (0.3 or 0.7 mg/kg every three weeks) for up to approximately 168 weeks. The main objective is to characterise long-term safety and tolerability, with additional exploratory endpoints addressing sustained haemodynamic, functional, and clinical effects in this population [114].

In addition, a local phase III study in Japanese PAH patients (MK-7962-020; NCT05818137) is ongoing [110]. This single-arm, open-label trial enrols mainly adults with WHO FC II–III PAH to confirm the efficacy and safety of sotatercept (0.7 mg/kg subcutaneously every three weeks for 24 weeks) in a Japanese population, thereby providing region-specific data that complement the global phase III programme [115].

The Phase 2 multicentre, single-blinded, randomized study MK-7962-024 (LIGHTRAY) trial (NCT06664801) is evaluating sotatercept (MK-7962) in adults with PAH receiving standard background therapy. Participants receive subcutaneous sotatercept every three weeks using a weight-based dosing strategy to assess safety, tolerability, and pharmacokinetics, and to compare weight-based with weight-banded dosing approaches. The study is expected to be completed in 2026, and its findings are intended to inform subsequent Phase 3 development of sotatercept in the PAH population [116].

The RECOMPENSE study (NCT06658522) is a prospective, single-arm, open-label Phase IV trial designed to assess the effects of sotatercept on right-ventricular function in adults with PAH receiving stable background therapy. The trial incorporates detailed haemodynamic evaluation and cardiac magnetic resonance imaging at baseline and Week 24 to quantify changes in RV structure, function, and RV–pulmonary artery coupling. Approximately 20 patients with idiopathic or heritable PAH are being enrolled, with sotatercept initiated at 0.3 mg/kg and increased to 0.7 mg/kg as tolerated. Study completion is anticipated in May 2026 [117].

Taken together, these ongoing and long-term extension studies extend the evidence base for sotatercept beyond the pivotal PAH trials, exploring its role across the disease spectrum—from paediatric PAH and high-risk adult PAH to Cpc-PH associated with HFpEF—and providing crucial data on durability of benefit and real-world-like safety with chronic exposure [110,118].

An overview of clinical trials evaluating sotatercept in pulmonary arterial hypertension is provided in Table 10 and Table 11. Table 10 summarises completed or terminated clinical trials, while Table 11 presents ongoing or recruiting studies.

### 4.5. Population Health Model Predicting the Impact of Sotatercept over a Lifetime Horizon

A comprehensive population-based health economic model developed by McLaughlin et al. [119] evaluated the long-term impact of adding sotatercept to standard background therapy in PAH. The analysis employed a six-state Markov structure, incorporating low-risk, intermediate–low-risk, intermediate–high-risk, high-risk, transplantation (lung or heart–lung), and death, enabling simulation of lifetime disease trajectories. Transition probabilities were derived from pivotal phase II/III sotatercept trials and large observational PAH registries, while post-transplant mortality estimates were sourced from published transplant survival data. All outcomes were discounted at 3% per year, and model robustness was evaluated through extensive deterministic and probabilistic sensitivity analyses. This modelling approach provided long-term projections of clinical outcomes and quantified the potential disease-modifying effects of sotatercept across the natural history of PAH.

Over a lifetime horizon, the addition of sotatercept was projected to increase mean life expectancy more than threefold relative to background therapy alone (16.5 vs. 5.1 years). Sotatercept was also associated with substantial reductions in the need for parenteral prostacyclin therapy, PAH-related hospitalizations, and transplantation procedures, underscoring its potential to substantially modify the long-term disease course and reduce overall healthcare burden associated with PAH [119].

### 4.6. Sotatercept and Specific Patient Groups

Pharmacokinetic analyses indicate that sotatercept exposure is not significantly influenced by demographic factors such as age (18–81 years), sex, or ethnicity. The overall safety profile of sotatercept was generally consistent across age subgroups, except for bleeding events, which were more frequently reported among patients aged ≥65 years compared with those younger than 65 years (52% vs. 31.9%). Despite this increased incidence, no clinically meaningful differences were observed in the type or severity of bleeding events between age groups.

The safety and efficacy of sotatercept in paediatric and adolescent populations (<18 years) remain unknown. The ongoing MOONBEAM study (ClinicalTrials.gov Identifier: NCT05587712) is expected to provide the first dedicated data on pharmacokinetics, safety, and clinical efficacy of sotatercept in children with PAH [111].

Sotatercept demonstrates body weight-dependent pharmacokinetics, with both clearance and central volume of distribution increasing proportionally with body mass The approved weight-based dosing regimen was developed to ensure uniform systemic exposure across patients.

In PAH patients with mild to moderate renal impairment (estimated glomerular filtration rate [eGFR] 30–89 mL/min/1.73 m^2^), sotatercept pharmacokinetics are comparable to those of individuals with normal renal function (eGFR ≥ 90 mL/min/1.73 m^2^). Available data from non-PAH populations indicate that end-stage renal disease does not significantly alter sotatercept disposition and that the drug is not dialysable. However, pharmacokinetic data in PAH patients with severe renal impairment (eGFR < 30 mL/min/1.73 m^2^) are lacking.

The pharmacokinetics of sotatercept in PAH patients with hepatic impairment (Child–Pugh classes A–C) have not been formally studied; however, clinically significant alterations in metabolism are not expected, as sotatercept is primarily cleared predominantly through non-enzymatic cellular catabolism rather than hepatic metabolism.

For women of childbearing potential, pregnancy testing prior to therapy and the use of effective contraception during treatment and for at least four months after the final dose are recommended. Clinical experience in pregnancy is absent. Preclinical reproductive toxicology studies have identified embryo-fetal developmental toxicity, including increased post-implantation loss, reduced fetal weight, and delayed skeletal ossification. On this basis, sotatercept is contraindicated during pregnancy and should not be administered to women of childbearing potential who are not using reliable contraceptive methods.

It is not known whether sotatercept or its metabolites are excreted into human breast milk. Therefore, breastfeeding is not advised during treatment and for a minimum of four months following the last administered dose to prevent potential exposure in nursing infants.

Preclinical studies also suggest that sotatercept may have the potential to impair fertility in both male and female animals, although the relevance to humans remains uncertain. No dedicated studies have yet been undertaken to evaluate the carcinogenic or mutagenic potential of sotatercept.

Sotatercept has been studied primarily as add-on therapy in patients with pulmonary arterial hypertension who failed to achieve or maintain a low-risk profile despite established standard-of-care treatments, representing a clinically high-risk population with insufficient response to conventional vasodilator-based strategies. While clinical trials demonstrate consistent benefit across major subgroups, further studies are required to formally characterise this population, define predictors of response, and optimise patient selection in real-world settings.

### 4.7. Sotatercept Within the Therapeutic Algorithm

Despite significant progress achieved with contemporary combination therapy and structured risk-based management, many patients with PAH continue to experience clinical deterioration, underscoring the persistent need for therapies that address the underlying remodelling biology of the disease [1,2,49,51,87]. Current guidelines advocate a risk-oriented therapeutic approach, in which management intensity is tailored to the estimated 1-year mortality risk, with the overarching objective of achieving and maintaining a low-risk profile—a condition strongly correlated with improved long-term survival [1].

Current recommendations from the 2022 ESC/ERS Guidelines and the proceedings of the 7th World Symposium on Pulmonary Hypertension place serial risk assessment at the centre of decision-making, integrating clinical status, exercise capacity, biomarkers, imaging, and haemodynamic variables to estimate 1-year mortality risk at diagnosis and during follow-up. While simplified risk models facilitate routine reassessment, both ESC/ERS and WSPH emphasise the value of complementary right-heart imaging and invasive haemodynamic, when feasible, to refine prognostic stratification and guide individualised treatment escalation [4,120].

**Figure 3:**
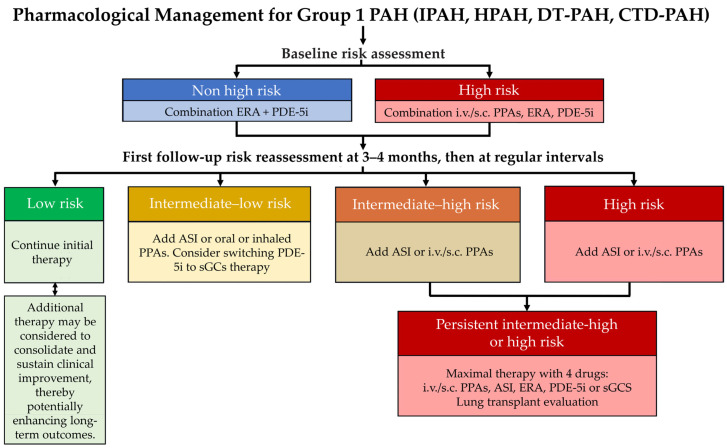
Pharmacological Management of Group 1 PAH (IPAH, HPAH, DT-PAH, and CTD-PAH), adapted by the Romanian Pulmonary Hypertension Expert Panel based on the proceedings of the 7th WSPH. ASI, activin signalling inhibitor; s.c., subcutaneous; CTD-PAH, connective tissue disease–associated pulmonary arterial hypertension; DT-PAH, drug- and toxin-induced pulmonary arterial hypertension; ERA, endothelin receptor antagonist; HPAH, heritable pulmonary arterial hypertension; PAH, pulmonary arterial hypertension; IPAH, idiopathic pulmonary arterial hypertension; i.v., intravenous; PDE-5i, phosphodiesterase-5 inhibitor, PPAs, prostacyclin pathway agents; sGCS, soluble guanylate cyclase stimulator.

Building on robust evidence from pivotal phase II–III trials, sotatercept, a first-in-class agent targeting dysregulated activin/GDF signalling within the TGF-β superfamily, has expanded the therapeutic landscape beyond conventional vasodilator pathways and supports the concept of disease modification in PAH. Reflecting this shift, the 7th WSPH incorporated sotatercept as an add-on option for patients who do not achieve or maintain a low-risk profile despite optimised background therapy [121]. In Romania, this approach has been adapted by the National Pulmonary Hypertension Expert Panel in alignment with local standards of care and resource availability (Figure 3).

Within this framework, sotatercept represents a mechanistically distinct add-on therapy that complements, rather than replaces, established vasodilator pathways. Unlike escalation within the endothelin, nitric oxide, or prostacyclin pathways, sotatercept targets upstream proliferative and remodelling processes and has been evaluated predominantly in patients who failed to achieve or maintain a low-risk profile despite optimised background therapy. In this context, sotatercept may be considered in patients with persistent intermediate or high risk despite dual or triple combination therapy, including as an alternative or adjunct to prostacyclin escalation in selected individuals. However, the optimal timing and prioritisation of sotatercept relative to parenteral prostacyclin initiation remain incompletely defined and will require further long-term and real-world evidence.

Practical considerations are essential when integrating sotatercept into routine clinical care. Treatment requires structured laboratory monitoring—particularly haemoglobin and platelet counts—consistent with on-target effects of activin/GDF–BMP pathway modulation, and this is especially relevant in patients receiving anticoagulation and/or parenteral prostacyclin. Although these monitoring requirements appear predictable and clinically manageable, they represent an additional operational consideration compared with most oral PAH therapies. Moreover, as a novel biologic agent, access, reimbursement, and cost are likely to vary across healthcare systems and may influence real-world uptake and positioning; accordingly, the proposed therapeutic framework should be regarded as evolving as experience accumulates.

Important knowledge gaps remain. Longer-term data are required to define the durability of benefit, optimal sequencing strategies, and the extent to which haemodynamic improvements translate into sustained right ventricular recovery and survival across PAH subgroups and comorbidity profiles. In parallel, further refinement of patient selection and monitoring strategies is needed, particularly in relation to mechanism-linked adverse events such as erythrocytosis, thrombocytopenia, and bleeding or telangiectasia. Finally, although no clinically meaningful pharmacokinetic differences by sex have been reported, sex-specific analyses remain highly relevant in PAH and should be systematically addressed in future trials and real-world registries to clarify potential differences in efficacy, safety, and monitoring requirements.

In conclusion, sotatercept represents a major advance in PAH therapeutics by targeting a central remodelling pathway and may contribute to a shift toward mechanism-based, risk-guided disease management alongside established vasodilator strategies.

## Figures and Tables

**Table 1:** Primary endpoint of the PULSAR trial: reduction in pulmonary vascular resistance with sotatercept (adapted from [96]).

Primary Endpoint	Placebo (*n* = 32)	Sotatercept 0.3 mg/kg (*n* = 32)	Sotatercept 0.7 mg/kg (*n* = 42)
PVR			
Change from baseline to week 24 (dyn·s·cm^−5^, mean ± SD)	116.4 ± 35.3 (−2.1%)	−162.2 ± 33.3 (−20.5%)	−255.9 ± 29.6 (−33.9%)

PVR, pulmonary vascular resistance; *n*, number of participants; SD, standard deviation. Percentage values indicate relative change from baseline.

**Table 2:** Secondary efficacy endpoints in the PULSAR trial: improvement in exercise capacity, cardiac function, and clinical status with sotatercept.

Secondary Endpoints	Placebo (*n* = 32)	Sotatercept 0.3 mg/kg (*n* = 32)	Sotatercept 0.7 mg/kg (*n* = 42)	All Sotatercept (*n* = 74)
6MWD (meters)				
LS mean difference compared with placebo (95% CI)	—	29.4 (3.8, 55.0)	21.4 (−2.8, 45.7)	24.9 (3.1, 46.6)
NT-proBNP (pg/mL)				
LS mean difference compared with placebo (95% CI)	—	−931.5 (−1353.2, −509.7)	−651.0 (−1043.3, −258.7)	−772.1 (−1125.2, −419.1)
TAPSE, cm				
LS mean difference compared with placebo (95% CI)		0.0 (−0.15, 0.15)	−0.1 (−0.2, 0.08)	−0.0 (−0.16, 0.09)
WHO-FC				
Participants with improvement of at least one FC, *n* (%)	4 (12)	10 (31)	7 (17)	17 (23)
Clinical worsening				
Participants with a clinical worsening event, *n* (%)	2 (6)	0	1 (2)	1 (1)

6MWD, 6 min walk distance; m, meters; CI, confidence interval; FC, functional class; *n*, number of participants; LS, least-squares; TAPSE, tricuspid annular plane systolic excursion; WHO-FC, World Health Organization functional class. Values are presented as least-squares (LS) mean differences versus placebo (95% confidence interval), derived from a mixed model for repeated measures adjusted for baseline covariates.

**Table 3:** Long-term efficacy outcomes from the PULSAR-OLE study: sustained improvements in pulmonary vascular resistance, exercise capacity, and biomarkers with continued sotatercept therapy.

	Placebo-Crossed Group					Continued-Sotatercept Group					
	Baseline	EOP	Months 18–24	Change from Baseline to Months 18–24	*p* Value	Baseline	EOP	Months 18–24	Change from Baseline to Months 18–24 ^#^	Change from EOP to Months 18–24	*p* Value ^†^
PVR (dyn·s·cm^−5^, mean ± SD)	802 ± 331	774 ± 355	583 ± 310	−223 ± 58	<0.0001	784 ± 372	564 ± 268	538 ± 199	−213 ± 254	−3 ± 159	0.8745
	(*n* = 30)	(*n* = 30)	(*n* = 25)	(*n* = 30) ^+,§^		(*n* = 67)	(*n* = 67)	(*n* = 57)	(*n* = 57)	(*n* = 57)	
6MWD (meters, mean ± SD)	409 ± 66	439 ± 85	480 ± 73	61 ± 13	<0.0001	398 ± 86	419 ± 87	458 ± 110	60 ± 81	7 ± 61	0.3987
	(*n* = 30)	(*n* = 30)	(*n* = 25)	(*n* = 30) ^+,§^		(*n* = 67)	(*n* = 67)	(*n* = 62)	(*n* = 62)	(*n* = 62)	
WHO-FC (numeric, mean ± SD)	2.4 ± 0.5	2.3 ± 0.5	1.9 ± 0.6	−0.6 ± 0.7	<0.0001	2.4 ± 0.5	2.0 ± 0.5	1.9 ± 0.5	−0.4 ± 0.6	−0.2 ± 0.5	0.0001
	(*n* = 30)	(*n* = 30)	(*n* = 28)	(*n* = 28)		(*n* = 67)	(*n* = 67)	(*n* = 63)	(*n* = 63)	(*n* = 63)	
NT-proBNP (pg/mL, mean ± SD)	840 ± 1247	1059.2 ± 1334.08	363 ± 702	−506.2 ± 1190	0.0004 *^f^*	777.4 ± 1051.03	777.4 ± 1051.03	268 ± 457	−470.5 ± 910.44	−76 ± 598	0.1384
	(*n* = 30)	(*n* = 30)	(*n* = 28)	(*n* = 28)		(*n* = 66)	(*n* = 66)	(*n* = 64)	(*n* = 63)	(*n* = 64)	

6MWD, 6 min walk distance; EOP, end of placebo-controlled treatment period; ƒ, nominal *p*-value; *n*, number of participants; NT-proBNP, N-terminal pro B-type natriuretic peptide; PVR, pulmonary vascular resistance; SD, standard deviation; SE, standard error, WHO-FC, World Health Organization functional class. All values are presented as mean ± SD unless otherwise indicated. #, all *p*-values < 0.0001; †, nominal *p*-values corresponding to change from end of placebo-controlled treatment period (EOP) to months 18–24; +: multiple imputation; §: values are mean ± SE.

**Table 4:** Secondary Clinical and Patient-Reported Outcomes in the STELLAR Trial (adapted from [15]).

	Placebo (N = 160)	Sotatercept (N = 163)	Sotatercept vs. Placebo	
	*n* (%)	*n* (%)		
Multicomponent improvement, n/N (%)	16 (10.1)	63 (38.9)	—	<0.001
PVR (dyn·s·cm^−5^, 95% CI)	—	—	−234.6 (−288.4 to −180.8)	<0.001
NT-proBNP (pg/mL, 95% CI)	—	—	−441.6 (−573.5 to −309.6)	<0.001
WHO-FC, n/N (%)	22 (13.8)	48 (29.4)	—	<0.001
Time to first occurrence of death or nonfatal clinical worsening event (95% CI)	—	—	0.16 (0.08 to 0.35)	<0.001
French low-risk score, n/N (%)	29 (18.2)	64 (39.5)	—	<0.001
PAH-SYMPACT Physical Impacts (95% CI)	—	—	−0.26 (−0.49 to −0.04)	0.01
PAH-SYMPACT Cardiopulmonary (95% CI)	—	—	−0.13 (−0.26 to −0.01)	0.028
PAH-SYMPACT Cognitive/Emotional (95% CI)	—	—	−0.16 (−0.40 to 0.08)	0.156

CI, confidence interval; N, total number of patients, *n*, number of patients who experienced a specific event or outcome; NT-proBNP, N-terminal pro B-type natriuretic peptide; PAH-SYMPACT, Pulmonary Arterial Hypertension—Symptoms and Impact Questionnaire; PVR, pulmonary vascular resistance; WHO-FC, World Health Organization functional class.

**Table 5:** STELLAR Trial—Distribution of First Clinical Worsening Events, Including Death, Hospitalization, or Transplantation Listing (data from [15]).

Risk of Death, Hospitalization, or Transplantation	Sotatercept (*n* = 163)	Placebo (*n* = 160)
Total number of patients who experienced at least one clinical worsening event or death, *n* (%)	9 (5.5%)	42 (26.3%)
Assessment of first occurrence of clinical worsening events or death, *n* (%):		
Death as first event	2 (1.2%)	6 (3.8%)
Worsening related listing for lung or heart transplant	1 (0.6%)	1 (0.6%)
Need to initiate rescue therapy or need to increase dose of infusion prostacyclin by 10% or more	2 (1.2%)	17 (10.6%)
PAH-related hospitalization (≥24 hrs)	0	7 (4.4%)
Deterioration of PAH	4 (2.5%)	15 (9.4%)

*n*, number of patients; PAH, pulmonary arterial hypertension.

**Table 6:** Safety Outcomes in the STELLAR Phase III Trial (adapted from [15]).

Number of Patients with any of the Following TEAEs	Placebo (N = 160) *n* (%)	Sotatercept (N = 163) *n* (%)
TEAEs of interest		
Bleeding events	72 (45.0)	97 (59.5)
Telangiectasia	25 (15.6)	52 (31.9)
Increased haemoglobin (increased haematocrit, increased RBC count)	6 (3.8)	23 (14.1)
Thrombocytopenia	0	10 (6.1)
Increased blood pressure	5 (3.1)	14 (8.6)
TEAEs with incidence ≥ 10% in one or more	1 (0.6)	7 (4.3)
Serious TEAEs related to treatment groups		
Epistaxis	3 (1.9)	33 (20.2)

N, total number of patients, *n*, number of patients who experienced TEAEs; RBC, red blood cell; TEAEs, treatment emergent adverse events.

**Table 7:** Primary Composite Endpoint in the ZENITH Phase III Trial (data from [17]).

Primary Endpoint	Sotatercept (*n* = 86)	Placebo (*n* = 86)
Composite of death from any cause, lung transplantation, or hospitalization for more than 24 h due to PAH worsening		
Number (%) of patients with ≥1 primary event	15 (17.4%)	47 (54.7%)
Components of the primary endpoint:		
All-cause death	7 (8.1%)	13 (15.1%)
Lung transplantation	1 (1.2%)	6 (7.0%)
PAH worsening-related hospitalization (≥24 h)	8 (9.3%)	43 (50.0%)

*n*, number of patients; PAH, pulmonary arterial hypertension.

**Table 8:** Secondary Clinical, Hemodynamic, and Patient-Reported Outcomes in the ZENITH Phase III Trial (adapted after [17]).

Secondary Endpoints	Sotatercept (*n* = 86)	Placebo (*n* = 86)
Overall survival (time-to-event analysis), (HR, 95% CI)	0.24 (0.13 to 0.43)	—
Transplant-free survival (time-to-event analysis), (HR, 95% CI)	0.42 (0.17 to 1.07)	—
Death from any cause	7 (8.1%)	13 (15.1%)
Median change from baseline in REVEAL Lite 2 risk score, (HR, 95% CI)	−3.0 (−3 to −2)	0.0 (0.0 to 0.0)
Patients with a low or intermediate REVEAL Lite 2 risk score (≤7) at week 24 (*n*, %)	34 (49.3%)	11 (15.3%)
NT-proBNP (pg/mL), (HR, 95% CI)	−1233.0 (−1233 to 1233)	255.4 (211 to 263)
Mean PAP, (HR, 95% CI)	−13.6 (−14 to −13)	5.5 (5 to 6)
PVR (dyn·s·cm^−5^), (HR, 95% CI)	−156.6 (−160 to −152)	46.6 (36 to 104)
WHO-FC (number/% of patients with improvement)	48 (55.8%)	24 (27.9%)
6MWD (meters), (HR, 95% CI)	45.4 (45.0 to 46.0)	−5.4 (−9.5 to −1.0)
CO (L/min), (HR, 95% CI)	−0.1 (−0.1 to −0.1)	−0.4 (−0.4 to −0.4)
EQ-5D-5L index score, (HR, 95% CI)	0.060 (−1.020 to 0.512)	0.007 (−0.358 to 0.740)

6MWD, 6 min walk distance; CI, confidence interval; CO, Cardiac output; EQ-5D-5L, EuroQol-5 Dimension 5 Level version; HR; hazard ratio; m, meters; *n*, number of patients; NT-proBNP, N-terminal pro B-type natriuretic peptide; PAP, pulmonary artery pressure; PVR, pulmonary vascular resistance; REVEAL, Registry to Evaluate Early and Long-term PAH Disease Management; WHO-FC, World Health Organization functional class. For 6MWD, CO, EQ-5D-5L score, NT-proBNP, mean PAP and PVR values represent median change estimate from baseline at week 24.

**Table 9:** Safety Outcomes in the ZENITH Phase III Trial. (data from [17]).

Adverse Events	Sotatercept (*n* = 86)	Placebo (*n* = 86)
Any AEs	85 (98.8%)	83 (96.5%)
AEs related to treatment	56 (61.1%)	28 (32.6%)
AEs leading to treatment discontinuation	0	4 (4.7%)
AEs leading to death	5 (5.8%)	12 (14.0%)
Severe AEs	32 (37.2%)	43 (50.0%)
Serious AEs	46 (53.5%)	55 (64.0%)
Serious AEs related to treatment	3 (3.5%)	2 (2.3%)
Selected AEs of interest or special interest		
Bleeding events	54 (62.8%)	30 (34.9%)
Serious bleeding events	5 (5.8%)	4 (4.7%)
Cardiac events	13 (15.1%)	26 (30.2%)
Increased haemoglobin	11 (12.8%)	1 (1.2%)
Telangiectasia	22 (25.6%)	3 (3.5%)
AEs with incidence ≥ 10% in one or more treatment groups		
Epistaxis	38 (44.2%)	8 (9.3%)
Telangiectasia	22 (25.6%)	3 (3.5%)
Gingival bleeding	9 (10.5%)	2 (2.3%)
Vomiting	11 (12.8%)	5 (5.8%)
Back pain	9 (10.5%)	4 (4.7%)

Adverse events (AEs) were defined as events with an onset occurring on or after the first dose of study treatment and up to 56 days after the last administered dose. The number of patients who died due to an AE differs from the number of deaths assessed in the primary and secondary efficacy endpoints, as the latter include all-cause mortality irrespective of attribution. AEs leading to death were evaluated within the safety set, which captures events occurring from the first dose through 56 days after the final dose of treatment.

**Table 10:** Completed or terminated clinical trials evaluating sotatercept.

Trial	Phase	Patients (*n*)	Population	Design	Primary Endpoint	Key Secondary Endpoints	Key Efficacy Outcomes	Key Clinical Outcomes	Safety/Adverse Effects
(Acronym, NCT)									
PULSAR	II	106	PAH, WHO-FC II–III	Randomized, double-blind, placebo-controlled	Change in PVR	6MWD; NT-proBNP; WHO-FC; hemodynamics	Significant reduction in PVR; improvement in 6MWD; NT-proBNP decrease	Improvement in WHO-FC; reduced clinical worsening	Increased hemoglobin; thrombocytopenia; epistaxis; telangiectasia
NCT03496207									
SPECTRA	IIa	21	PAH, WHO-FC III	Open-label, single-arm	Change in peak VO_2_	RV volumes and function (cMRI); ventilatory efficiency	Increased peak VO_2_; improved RV structure and function	Evidence of RV reverse remodeling	Hematologic abnormalities; bleeding risk
NCT03738150									
STELLAR	III	323	PAH, WHO-FC II–III	Randomized, double-blind, placebo-controlled	Change in 6MWD at 24 weeks	PVR; NT-proBNP; WHO-FC; time to clinical worsening; QoL	+40.1 m improvement in 6MWD; reduced PVR and NT-proBNP	84% reduction in death or clinical worsening	Epistaxis; gingival bleeding; telangiectasia; increased hemoglobin
NCT04576988									
ZENITH	III	172	High-risk PAH, WHO-FC III–IV	Randomized, double-blind, placebo-controlled	Time to morbidity/mortality	NT-proBNP; PVR; mPAP; 6MWD; transplant-free survival	Improvement in hemodynamics and biomarkers	76% reduction in morbidity and mortality	Bleeding events; erythrocytosis; thrombocytopenia
NCT04896008									
HYPERION	III	320	Newly diagnosed PAH, intermediate/high risk	Randomized, double-blind, placebo-controlled	Time to clinical worsening	Risk score evolution; NT-proBNP; WHO-FC; 6MWD	Early and sustained biomarker and functional improvement	76% reduction in clinical worsening	Hematologic abnormalities; bleeding events
NCT04811092									
CADENCE	II	142	Cpc-PH due to HFpEF	Randomized, double-blind, placebo-controlled	Change in PVR at Week 24	6MWD; NT-proBNP; RV function	Significant reduction in PVR	Improvement in functional status	Hematologic effects; bleeding risk
NCT04945460									

6MWD, 6 min walk distance; cMRI, cardiac magnetic resonance imaging; Cpc-PH, Combined Pre- and Post-Capillary Pulmonary Hypertension; HFpEF, heart failure with preserved ejection fraction; Key efficacy outcomes, predefined trial outcomes assessing treatment-related efficacy (e.g., hemodynamic, functional, or biomarker-based measures); Key clinical outcomes, clinically meaningful outcomes including clinical worsening, hospitalization, transplantation, or death; mPAP, mean pulmonary arterial pressure; NCT, National Clinical Trial (identifier); NT-proBNP, N-terminal pro–B-type natriuretic peptide; PAH, pulmonary arterial hypertension; PVR, pulmonary vascular resistance; QoL, quality of life; RV, right ventricle/right ventricular; VO_2_, maximum volume of oxygen a body can use during intense exercise; WHO-FC, World Health Organization functional class.

**Table 11:** Ongoing or recruiting clinical trials evaluating sotatercept.

Trial	Phase	Patients (*n*)	Population	Design	Primary Endpoint	Key Secondary Endpoints	Key Efficacy Outcomes	Key Clinical Outcomes	Safety/Adverse Effects
(Acronym, NCT)									
SOTERIA	III (extension)	815	PAH completing parent trials including HYPERION	Long term, open-label extension	Long-term safety	Durability of efficacy; functional and biomarker outcomes	Sustained improvements in functional and biomarker measures	Maintenance of low-risk clinical profile	Long-term safety; bleeding; hematologic abnormalities
NCT07218029									
HARMONIZE	II (extension)	130	Participants from CADENCE	Double-blind extension	Long-term safety	Sustained hemodynamic and functional outcomes	Sustained efficacy signals over time	Maintenance of clinical stability	Long-term safety monitoring
NCT06814145									
RECOMPENSE	II	20	PAH with RV dysfunction	Open-label interventional	Change in RV compensation	RV functional parameters; exercise capacity	Improvement in RV functional metrics	Exploratory improvement in exercise tolerance	Safety and tolerability assessments
NCT06658522									
Japanese PAH study	III	46	Japanese adult PAH	Open-label, add-on sotatercept	Change in PVR at week 24	6MWD; WHO-FC; NT-proBNP	Reduction in PVR and NT-proBNP	Improvement in WHO-FC and exercise capacity	Hematologic monitoring; bleeding events
NCT05818137									
MOONBEAM	II	50	Pediatric PAH (1–17 years)	Open-label, single-arm	Safety and pharmacokinetics	Exploratory biomarkers; hemodynamics	PK characterization and exploratory efficacy	Not powered for clinical outcomes	Safety and developmental monitoring
NCT05587712									
Central cardiopulmonary performance study	II	21	Adult PAH	Interventional	Change in cardiopulmonary performance	Exercise and RV functional parameters	Improvement in cardiopulmonary metrics	Exploratory functional improvement	Safety and tolerability
NCT06409026									

6MWD, 6 min walk distance; HFpEF, heart failure with preserved ejection fraction; NCT, National Clinical Trial (identifier); NT-proBNP, N-terminal pro–B-type natriuretic peptide; PAH, pulmonary arterial hypertension; PK, pharmacokinetics; PVR, pulmonary vascular resistance; RV, right ventricle/right ventricular; WHO-FC, World Health Organization functional class.

## Data Availability

No new data were created or analysed in this study. Data sharing is not applicable to this article.

## Abbreviations

The following abbreviations are used in this manuscript:

6MWD	6 min walk distance
7th WSPH	7th World Symposium on Pulmonary Hypertension
ActRIIA	activin receptor type IIA
ADA	anti-drug antibody
AE	adverse event
AESI	adverse event(s) of special interest
BMPs	bone morphogenetic proteins
BMPR2	bone morphogenetic receptor type 2
BNP	B-type natriuretic peptide
CI	confidence interval
Cmax	maximum plasma concentration
cMRI	cardiac magnetic resonance imaging
CO	cardiac output
Cpc-PH	combined post-capillary and pre-capillary pulmonary hypertension
EC	endothelial cell
ECM	extracellular matrix
eGFR	estimated glomerular filtration rate
EMA	European Medicines Agency
EndMT	endothelial-to-mesenchymal transition
EOP	end of placebo-controlled treatment period
EQ-5D-5L	EuroQol-5 Dimension 5 Level (quality-of-life questionnaire)
ERS	European Respiratory Society
ESC	European Society of Cardiology
FC	functional class
FDA	Food and Drug Administration
GDFs	growth differentiation factors
HFpEF	heart failure with preserved ejection fraction
HR	hazard ratio
mPAP	mean pulmonary artery pressure
NNT	number needed to treat
NT-proBNP	N-terminal pro B-type natriuretic peptide
PAH	pulmonary arterial hypertension
PAH-SYMPACTs	PAH-SYMPACT assessment questionnaires
PAP	pulmonary artery pressure
PASMC	pulmonary artery smooth muscle cell
PAWP	pulmonary arterial wedge pressure
PH	pulmonary hypertension
PVR	pulmonary vascular resistance
RBC	red blood cell
REVEAL	Registry to Evaluate Early and Long-term PAH Disease Management
REVEAL Lite 2	simplified REVEAL risk score (REVEAL Lite 2 risk score)
RV	right ventricle
TAPSE	tricuspid annular plane systolic excursion
TEAE	treatment-emergent adverse event
TGF-β	transforming growth factor beta
Tmax	time to peak concentration
TTCW	time to clinical worsening
WHO-FC	World Health Organization functional class
WU	Wood units
